# PCBP-1 Regulates the Transcription and Alternative Splicing of Inflammation and Ubiquitination-Related Genes in PC12 Cell

**DOI:** 10.3389/fnagi.2022.884837

**Published:** 2022-06-20

**Authors:** Aishanjiang Yusufujiang, Shan Zeng, Chen Yang, Sha Jing, Lijuan Yang, Hongyan Li

**Affiliations:** ^1^Department of Neurology, People’s Hospital of Xinjiang Uygur Autonomous Region, Ürümqi, China; ^2^Xinjiang Clinical Research Center for Stroke and Neurological Rare Disease, Ürümqi, China

**Keywords:** PCBP1, alternative splicing, Parkinson’s disease, LCN-2, WWP-2

## Abstract

PCBP-1, a multifunctional RNA binding protein, is expressed in various human cell/tissue types and involved in post-transcriptional gene regulation. PCBP-1 has important roles in cellular Iron homeostasis, mitochondrial stability, and other cellular activities involved in the pathophysiological process of neurodegenerative diseases, such as amyotrophic lateral sclerosis (ALS) and Huntington’s disease (HD). However, it remains enigmatic whether PCPB-1 is associated with the pathogenesis of PD. In this study, we cloned and constitutively overexpressed PCBP-1 in rat PC12 cells (PC12 cell is the common cell line studying neurodegenerative disease include PD). RNA-seq was performed to analyze PCBP-1-regulated differentially expressed genes (DEGs) and alternative splicing events (ASEs) between control and PCBP1-overexpressed cells. GO and KEGG pathway analyses were performed to identify functional DEGs and alternatively spliced genes. Consequently, we validated PCBP-1-regulated genes using RT-qPCR. Finally, we downloaded CLIP-seq data from GEO (GSE84700) to analyze the mechanisms of PCBP-1’s regulation of gene expression and ASEs by revealing the binding profile of PCBP-1 on its target pre-mRNAs. Overexpression of PCBP-1 partially regulated the ASE and expression of genes enriched in neuroinflammation and protein ubiquitination, which were also associated with PD pathogenesis. Moreover, RT-qPCR assay verified the PCBP-1-modulated expression of neuroinflammatory genes, like *LCN-2*, and alternative splicing (AS) of ubiquitination-related gene *WWP-2*. Finally, CLIP-seq data analysis indicated that the first UC motif was the critical site for PCBP-1 binding to its targets. In this study, we provided evidence that PCBP-1 could regulate the expression of *LCN-2* gene expression associated with neuroinflammation and AS of WWP-2 in relation to protein ubiquitination. These findings thus provided novel insights into the potential application of PCBP-1 as the disease pathophysiological or therapeutic target for neurodegenerative disease.

## Introduction

Parkinson’s disease (PD), a progressive neurodegenerative condition primarily affecting the midbrain, is the second most leading cause of neurodegenerative disorder in the elderly population, following Alzheimer’s disease (AD), while the etiological factors largely remain unknown (Tysnes and Storstein, 2017). To date, genetic mutation is considered the most common etiology of PD pathogenesis. However, pathogenic variations in 20 monogenic loci can only account for 10–20% of PD onsets (Blauwendraat et al., 2020). In addition, the transcriptional and post-transcriptional regulations also play important roles in PD pathophysiology (Blackinton et al., 2009; Boon, 2016; Sato et al., 2021). Given that RNA binding proteins (RBPs) are key post-transcriptional regulators, any abnormalities in this family of proteins can modulate the pathophysiology of the neurodegenerative diseases through regulating the disease-associated gene expression (Brinegar and Cooper, 2016; Conlon and Manley, 2017; Sidibé et al., 2021). For example, TAR DNA binding protein of 43 kDa (TDP-43) is involved in apoptosis, cell division, and axonal transport through regulation of transcription, alternative splicing, and mRNA stability in frontotemporal dementia (FTD), AD, and amyotrophic lateral sclerosis (ALS; Hanson et al., 2012; Jo et al., 2020; Klim et al., 2021).

RBPs bind to their respective target RNAs through highly conserved RNA-binding domains (RBDs) in order to regulate the functionality of those RNAs. Poly (rC)-binding protein 1 (PCBP-1), a key member of the PCBP family of proteins, is widely expressed in various human tissue types. PCBP-1 plays an important role in the regulation of transcription and RNA metabolism at various levels (Geuens et al., 2017). The previous studies have also indicated that PCBP-1 regulates the circadian clock (Wu et al., 2021) as well as maintains the iron homeostasis (Yanatori et al., 2020). Furthermore, abnormal nuclear distribution of PCBP-1 participates in Huntington’s disease (HD) pathogenesis, suggesting that as RBPs, like PCBP-1, may contribute to the pathophysiological processes under neurodegenerative conditions (Geuens et al., 2017; Mori et al., 2019). Since PD and HD share common pathophysiology, PCBP-1 may be closely associated with PD as well, but the exact mechanism is not yet clear (Artyukhova et al., 2019; McLeary et al., 2019).

Up to now, most *in vitro* studies related to neurodegenerative diseases were performed in PC12 (rat pheochromocytoma) cells because of their inherent capacity to produce neuronal synapses and express key neuronal proteins (Su and Shih, 2015; Yang T.-C. et al., 2016; Zhang et al., 2016; Lee et al., 2018; Wilkaniec et al., 2019). In this study, we genetically modified PC12 cells derived from the pheochromocytoma of the rat adrenal medulla by stably overexpressing PCBP-1. We then performed RNA sequencing analysis to delineate the potential roles of PCBP-1 in modulating transcription and pre-mRNA alternative splicing (AS) related to neuroinflammation, apoptosis, endocytosis, and protein ubiquitination. Then, comparative analyses were carried out to highlight the different roles of PCBP-1 in gene activation and AS. Our findings thus indicate that PCBP-1 acts as the essential modulator of neuroinflammation, post-translational protein ubiquitination, and AS in the PD pathophysiology.

## Materials and Methods

### Plasmid

The EGFP-tagged PCBP-1 overexpression plasmid pCDNA3.1-PCBP1-EGFP-C2 was purchased from (YouBio Technology Co., Ltd., Xi’an, China).

### Cell Culture and Transfections

PC-12 cells (Procell Life Sciences, China) were cultured in RPMI-1640 medium, supplemented with 10% fetal bovine serum (FBS), 50 μmol/L β-mercaptoethanol, 100 U/mL penicillin, and 100 μg/mL streptomycin with 5% CO_2_ at 37°C. PCBP-1 plasmid was transfected in PC-12 cells by Lipofectamine-2000 reagent (Invitrogen, United States), according to the manufacturer’s protocol, and cells were harvested at 48 h post-transfection for RT-qPCR analysis.

### Gene Expression Analysis

Glyceraldehyde-3-phosphate dehydrogenase (GAPDH) was the house-keeping control for PCBP-1 overexpression analysis. cDNA synthesis and RT-qPCR assay were done using Bestar SYBR Green RT-PCR Master Mix (DBI Bioscience, China) and Bio-Rad S1000 machine. The primer sequences are listed in the Supplementary Table 1. The normalized mRNA levels were calculated by 2^–ΔΔ^ *^Ct^* method (Livak and Schmittgen, 2001), and paired Student’s *t*-test was performed for comparative analysis in GraphPad Prism.

### RNA Extraction and Sequencing

RQ1 DNase (M610A, Promega) was used to remove genomic DNA contamination from total RNA prior to construction of directional RNA-seq library by KAPA mRNA-Seq Kit (KK8541, Roche). RNA concentration and quality were measured using NanoDrop 2000 (Thermo, China). 1.5% Agarose gel electrophoresis was employed to check RNA integrity.

Poly-A tailed mRNAs were purified from 1 μg of total RNA and fragmented, followed by conversion into respective cDNA. Then, Roche DNA Adaptors (8005702001, Roche) were ligated to end-repaired and A-tailed double-stranded (ds) cDNA. Ligation products corresponding to 300–500 bps were then processed and stored at −80°C for downstream applications. dUTP incorporated cDNA strand was not amplified for strand-specific sequencing.

Illumina Novaseq 150 nt paired-end kit was applied for cDNA library preparation for high-throughput sequencing.

### Raw Data Cleaning and Alignment

2-N bases containing raw reads and reads with less than 16 nt were first eliminated, followed by FASTX-Toolkit v0.0.13 guided trimming of adaptor sequences and low-quality bases. The Ensemb Rat genome (Rnor_6.0) alignment of the clean reads was performed by TopHat2 (Kim et al., 2013), allowing 4 mismatches maximum. Thus obtained uniquely mapped reads were then used for the fragments per kilobase of transcript per million fragments mapped (FPKM) calculation and counting gene read numbers (Trapnell et al., 2010).

### Analysis of Differentially Expressed Genes

Differentially Expressed Genes (DEGs) were screened by R Bioconductor package edgeR (Robinson et al., 2010) by applying the false discovery rate (FDR) < 0.05, and fold change > 2 or <0.5 as the cut-off.

### Alternative Splicing Analysis

Alternative splicing (AS) and regulated AS (rAS) events were classified by identification of 10 types of splice-junction events, including alternative 5′ splice site (A5SS), exon skipping (ES), alternative 3′splice site (A3SS), mutually exclusive exons (MXE), intron retention (IR), mutually exclusive 5′UTRs (5pMXE), mutually exclusive 3′UTRs (3pMXE), and cassette exon using the ABLIRC pipeline (Xia et al., 2017). To assess RBP-mediated rAS events, Student’s *t*-test was performed, and the events with significant *P*-value and FDR cut-off were finally considered.

### RT-qPCR Validation of Differentially Expressed Genes and Alternative Splicing Events

The primers used for individual DEG validation using RT-qPCR assay are listed in Supplementary Table 1. Remainders of total RNA were used for cDNA preparation using M-MLV RT enzyme (Vazyme, China), followed by RT-qPCR assay using One Step RT-PCR System (Yepsen). The PCR conditions used were as follows: denaturation at 95°C for 10 min, then 40 cycles of denaturation at 95°C for 15 s, annealing and extension at 60°C for 1 min. Reactions for each sample were performed in triplicate and normalized against GAPDH.

In addition, AS and rAS events were validated by qRT-PCR assay. The primer sequences are shown in the Supplementary Table 1. The boundary-spanning primers were employed for detecting alternatively spliced mRNA isoforms.

### Functional Enrichment Analysis

Gene Ontology (GO) terms and KEGG pathway analyses were carried out to functionally categorize the identified DEGs using KOBAS 2.0 server (Xie et al., 2011). Further, enrichment of each GO term was defined by the hypergeometric test and Benjamini-Hochberg FDR controlling procedure.

### Analysis of PCBP-1 Binding to Its Target Genes Using CLIP-Seq Data

To further clarify the target genes of PCBP-1, we downloaded and reanalyzed the Clip-seq data of PCBP-1 from GEO (GSE84700) in Jurkat T cells as described earlier (Wang et al., 2018), sequencing library preparation was done, and 150-nt paired-end sequencing was performed on the Illumina HiSeq X Ten system.

A computational simulation was used to randomly generate reads similar in number and length as in peak and reads with at least 1 bp were clustered at peaks. The overlapping output reads were then mapped back to the respective gene to generate random max peak height, repeatedly for 500 times. The peak heights which were higher than random max peaks (*P* < 0.05) were selected. The PCBP-1 peaks overlapping with the input peaks were detected and eliminated by independent simulation analysis. The PCBP-1 target genes were determined by analyzing the locations of PCBP-1 binding peaks on the Rat genome by calling binding motifs of PCBP-1 by Homer software. Finally, we performed overlapping analysis for GSE84700 CLIP-seq data with PCBP-1 overexpression using ABLIRC analysis methods.

## Results

### Profiling of PCBP-1-Regulated Gene Expression

**FIGURE 1:**
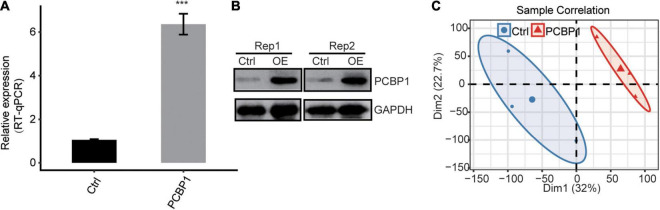
PCBP-1 expression quantified by **(A)** qRT-PCR, **(B)** western blotting, **(C)** PCA of PCBP1-OE and control, based on FPKM values. Error bars represent mean ± SEM. ***P < 0.001.

Here, we exploited the PCBP-1 overexpression (PCBP-1-OE) model in PC12 cells to investigate PCBP-1’s role in gene regulation. *PCBP-1* gene expression level was significantly higher in PCBP1-OE compared to the control (Figure 1A). In addition, PCBP-1 protein level was higher in PCBP1-OE cells (Figure 1B). The six RNA-seq libraries were constructed from PCBP1-OE cells for transcriptional profiling on the Illumina Novaseq system. After validating the reliability of RNA-seq data, FPKM was computed for gene expression variation across samples. RNA-seq assessment was performed for PCBP-1 overexpression. FPKM values of all expressed genes were used to compute the principal component analysis (PCA) for the identification of the global expression patterns of six samples. Consequently, the presentation of the top two components revealed the clear separation between the control and PCBP1-OE cells (Figure 1C). Significant association of the biological replicates indicated the quality and reliability of the RNA-seq data and that PCBP-1 overexpression could alter the transcriptome profiles.

### PCBP-1 Up-Regulates Genes Enriched in Inflammation Response in PC12 Cells

**FIGURE 2:**
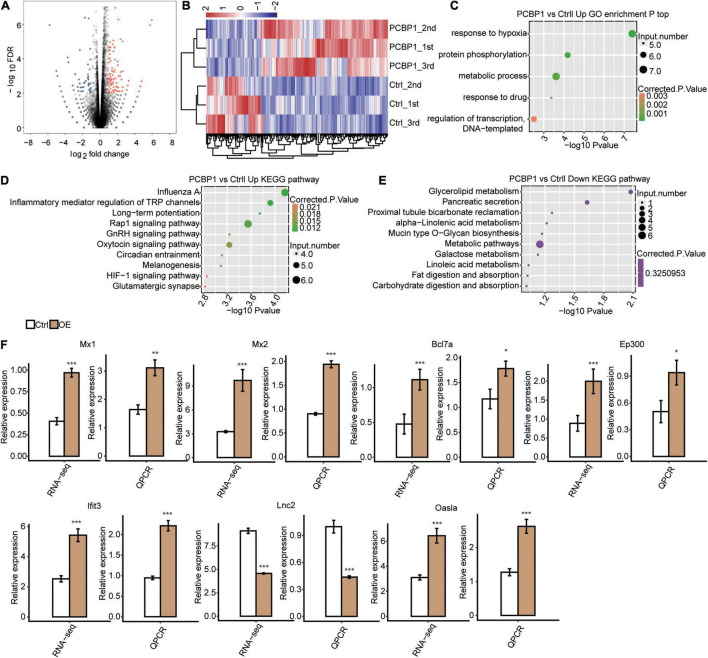
RNA-seq analysis of PCBP-1 regulated transcriptome profile. **(A)** Identification of PCBP-1 regulated genes. Up-regulated genes are labeled in red, down-regulated ones are in blue in the volcano plot. **(B)** Hierarchical clustering of DEGs in PCBP1-OE and control samples. FPKM values were log2-transformed and then median-centered by each gene. **(C)** The top 10 representatives GO biological processes pathways for up-regulated genes. **(D)** The top 10 representative KEGG pathways for up-regulated genes. **(E)** The top 10 representative KEGG pathways for downregulated genes. **(F)** Inflammatory response-related gene expression regulation by PCBP-1. Error bars represent mean ± SEM. *P < 0.05, **P < 0.01, ***P < 0.001.

For investigation of the PCBP-1’s role in the post-transcriptional regulations, we analyzed the RNA-seq data of PCBP1-OE and control samples by adjusting absolute fold change ≥ 2 and FDR ≤ 0.05 with the edgeR package and determined the DEGs (Supplementary Table 2). Subsequently, we identified 132 up-regulated and 58 downregulated genes in PCBP1-OE cells by constructing the volcano plot (Figure 2A). Furthermore, heat map estimation confirmed the DEG expression trends as observed in RNA-seq analysis in PCBP1-OE cells (Figure 2B).

### GO and KEGG Analyses

To understand the functional roles of PCBP-1 in PC12 cells, KEGG pathway and GO enrichment analyses were carried out. GO analysis showed that responses to hypoxia, protein phosphorylation, metabolic process, response to the drug, regulation of transcription DNA-templated at the molecular function (MF) level were enriched (Figure 2C). Moreover, KEGG analysis exhibited upregulation of DEGs primarily in pathways, including Influenza A, inflammation-mediated regulation of TRP channels, long-term potentiation, Rap1 signaling pathway, GnRH signaling pathway, oxytocin signaling pathway, circadian entrainment, melanogenesis, HIF-1 signaling pathway, and glutamatergic synapse formation (FDR corrected *P* ≤ 0.05, Figure 2D). Furthermore, the downregulated DEGs were mostly enriched in glycerolipid metabolism, pancreatic secretion, proximal tubule bicarbonate reclamation, alpha-linolenic acid metabolism, mucin-type O-glycan biosynthesis, metabolic pathways, galactose metabolism, metabolism of linoleic acid, and digestion and absorption of fat and carbohydrate pathways (Figure 2E). Amongst them, the inflammatory pathway, Rap1 signaling pathway, and circadian entrainment are closely associated with PD (Yang X. Z. et al., 2016; Maggio et al., 2019).

We found that PCBP-1 overexpression activated the neuroinflammation-related gene expression. Furthermore, we conducted RT-qPCR analysis to individually assess the levels of lipocalin-2(LCN2), Mx1, Mx2, Bcl7a, Ep300, Lfit3, and Oasla mRNAs. Previous studies have shown that (lipocalin-2) LCN2 are highly expressed in neuroinflammation (Mondal et al., 2020; Lim et al., 2021). Our results revealed a significant decrease in LCN2 expression with a simultaneous increase in Mx1, Mx2, Bcl7a, Ep300, Lfit3, and Oasla expressions, which were significantly consistent with the sequencing results (Figure 2F).

### PCBP-1 Selectively Modulates the Alternative Splicing of Genes Involved in Protein Ubiquitination

**FIGURE 3:**
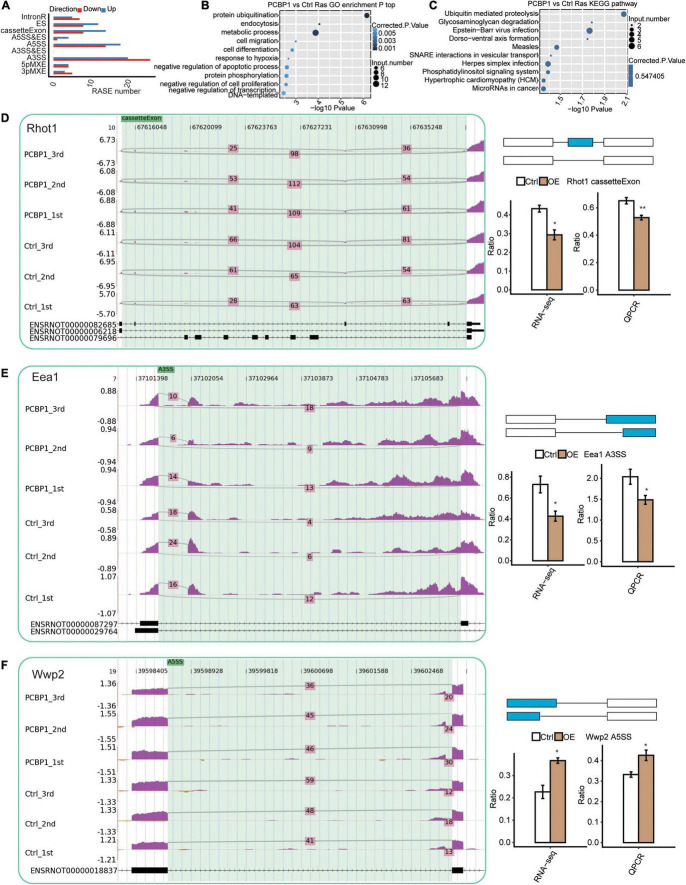
Identification and functional analysis of PCBP-1-mediated AS events. **(A)** Classification of different PCBP-1 regulated AS events. **(B)** The top 10 GO biological process analyses and **(C)** KEGG functional pathway of the alternatively spliced genes. PCBP-1 regulated AS of genes involved in ubiquitination and endocytosis, including **(D)** Rab1a, **(E)** Rhot1, and **(F)** Eea1. Left panel: The schematic diagrams depicting the structures of ASEs, AS1 (purple line), and AS2 (green line). Right panel: The constitutive exons are denoted by black boxes, intron sequences by a horizontal line (top), while alternative exons by a red box. The bottom right panel represents RNA-seq and RT-qPCR validation of AS events. Error bars represent mean ± SEM. **P < 0.01, *P < 0.05.

We assessed the RNA-seq data quality for differential splicing in PC12 cells. A total of 49.4 ± 1.5 million uniquely mapped reads were retrieved from both samples, including 33.5% junction reads. The RNA-seq data detected 268438 novel splice junctions and 174170 annotated exons (Figure 3A and Supplementary Table 3). Subsequently, the ABLas software (Xia et al., 2017) was utilized to assess the AS events in the global variations of the AS profiles in PCBP1-OE. The results showed that PCBP-1 regulates hundreds of ASEs in PC12 cells. In the GO assessments, the alternatively spliced genes were enriched in protein ubiquitination, endocytosis, metabolic process, cell migration, cell differentiation, response to hypoxia, negative regulation of the apoptotic process, protein phosphorylation, negative regulation of cell proliferation, and transcription DNA-templated pathways in PCBP1-OE cells (Figure 3B). Enriched KEGG pathways included ubiquitin-mediated proteolysis, glycosaminoglycan degradation, Epstein-Barr virus infection, Dorsoventral axis formation, measles, SNARE interactions in vesicular transport, herpes simplex infection, phosphatidylinositol signaling system, hypertrophic cardiomyopathy, and microRNAs in cancer (Figure 3C). Thus PCBP-1 potentially played a critical role in protein ubiquitination, neuroinflammation, endocytosis, and immunity by regulating AS of associated genes. Then, we used the RT-qPCR assay to examine the changes of the type of AS between control and PCBP1-OE cells. Figures 3D–F exhibits representative RAS events, including cassette exon, alternative 3′splice site, and alternative 5′splice site (Supplementary Table 4). Among those alternatively spliced genes, dysregulation of Rhot1, Eea1, and Wwp2 was associated with mitochondrial damage, endocytosis, and protein ubiquitination. To obtain further insight into RAS events of Rhot1, Eea1, and Wwp2, we used RT-qPCR to confirm if these changes were related to PCBP-1 overexpression in PC12 cells. As shown in Figures 3D–F, the AS events of Rhot1 and Eea1 showed a decreasing trend, whereas Wwp2 exhibited an increase. Together, our results suggested that AS of Rhot1, Eea1, and Wwp2 were regulated by PCBP-1 directly.

### Functional Analysis of the PCBP-1 Bound Genes in PC12 Cells

**FIGURE 4:**
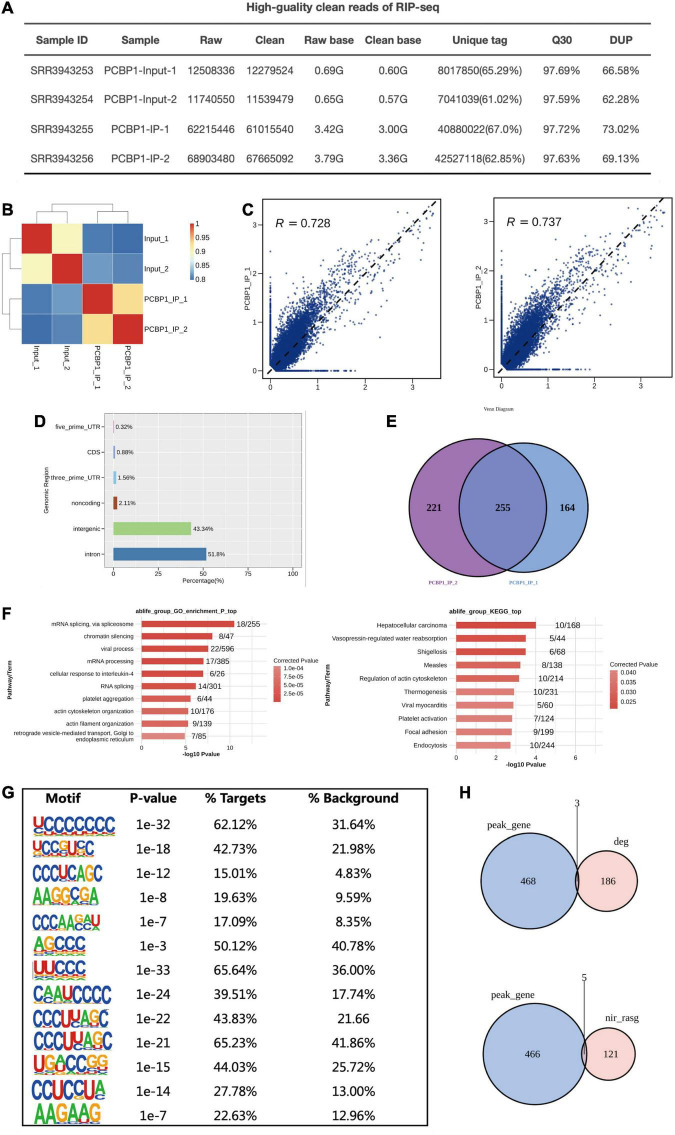
PCBP-1 shows a strong association with RNAs from intergenic and intron regions. **(A)** The quality control of the CLIP sequence was good. (1) Raw data: the number of original sequences transformed from the original image data obtained by sequencing through base calling; (2) Clean reads: the raw reads were trimmed of the adapter sequences, and the number of valid sequences obtained after low-quality bases were used for subsequent analyses; (3) Raw base: the count of the number of bases it contained, based on the number and length of raw reads, in G; (4) Clean base: according to the number and length of clean reads, count of the number of bases it contained, in G; (5) Unique tag: unique tag, the number of non-repeating reads and its proportion of clean reads; (6) Q30: Proportion of bases whose sequencing error rate was less than 0.1%; (7) DUP: duplication level. The ratio of duplicate reads to total reads. **(B,C)** Heat map showing the hierarchically clustered Pearson’s correlation matrix resulting from a comparison of the transcript expression values for control and PCBP-1 immunoprecipitation (IP) samples. **(D)** Bar plot of the genomic region distribution of the control and uniquely mapped PCBP-1 IP reads. **(E)** Venn diagram analysis from the comparative results of ABLIRC peak calling methods. **(F)** GO and KEGG analyses from the comparative results. **(G)** Extracted PCBP1 peaks motifs using ABLIRC Methods. **(H)** Venn diagram from overlapping analysis results for GSE84700 CLIP-seq data with PCBP1 OE RNA-seq using ABLIRC analysis methods.

The ABLIRC tool was applied to elucidate the PCBP-1 target genes from the CLIP-seq reads downloaded from GEO (GSE84700). As displayed in Figure 4A, we can see that the quality control of CLIP-sequence is good. The correlation of PCBP1 samples was high and obviously different from that of the control (Figure 4B). A plot of the assayed levels of each gene was reflected using the FPKM in each pair of samples, which suggested that transcripts were enriched in the IP samples (Figure 4C). The distribution of uniquely mapped anti-PCBP1 reads was compared to the overall human genome, and the CLIP-seq reads were much more highly enriched in intron and intergenic regions (Figure 4D). The peaks were identified using the ABLIRC algorithm. Peaks from the two sets of experiments overlapped well (Figure 4E). GO enrichment analysis indicated that these genes interacting with PCBP1 were highly enhanced for modulation of mRNA splicing, chromatin silencing, mRNA processing, and RNA splicing (Go biological process terms, Figure 4F, left panel). Enriched KEGG cascades constituted regulation of actin cytoskeleton, focal adhesion, and endocytosis (Figure 4F, right panel). The UC-rich motifs were highly enriched in the AS overlapping PCBP-1 bound peaks (Figure 4G), suggesting that the first UC motifs were the important target sites for PCBP-1. Consequently, 5 of the target genes overlapped with the PCBP-1-regulated alternatively spliced genes (121) (Figure 4H and Supplementary Table 5).

## Discussion

In this study, we found that the RNA-binding protein PCBP-1 could mediate the transcriptional and post-transcriptional activities of its several target genes in PC12 cells. However, the underlying regulatory mechanisms were largely unknown. To the best of our knowledge, we established that PCBP-1 partially modulated the differential expression of genes associated with neuroinflammation and neuroimmune mechanisms in PC12 cells, involving 132 up-regulated and 58 downregulated genes, indicating PCBP-1 expression level is critical in the neuronal cells.

As an iron-trafficking protein, LCN-2 was the most important gene, down-regulated by PCBP-1 overexpression contributing to both oxidative stress and inflammation. Recently, LCN-2 has been reported to possess various roles in cellular iron transportation and homeostasis. In the central nervous system (CNS), LCN-2 is known to play a role in neuroinflammation under neurodegeneration, glioma, brain injury, and multiple sclerosis conditions (Devireddy et al., 2005; Bao et al., 2010; Bhusal et al., 2021). Byung-Wook Kim has reported that LCN-2 expression can increase in the substantia nigra pars compacta (SNpc) in patients with PD (Kim et al., 2016). Consistently, there were also several up-regulated genes that were involved in various immune processes, including mycovirus (influenza virus) resistance 1 (Mx1), MX dynamin-like GTPase 2 (Mx2), and Bcl7a. Bcl7a has been described as B-cell CLL/lymphoma7 protein family member A, and it is involved in PD through the TNF-α and NF-κB signaling axis (Wischhof et al., 2017). The genetic deletion of Bcl7a in postmitotic neurons elicits motor abnormalities (Lechner et al., 2021). These findings provide novel proof for neuroinflammation and neuroimmune as the crucial modulators for PD pathophysiology.

RNA-seq analysis showed that PCBP-1 partially modulated AS in PC12 cells. Particularly, PCBP-1 binding was associated with increased RASE numbers. Functionally, these changes in gene expression patterns impacted protein ubiquitination and endocytosis. Specifically, PCBP1 correlated with protein modulation. Notably, ubiquitin signaling plays a pivotal role in protein quality control. Ubiquitinylated and misfolded proteins are aggregated in PD, which includes genes encoding regulatory molecules, such as PTEN-induced kinase 1 (PINK1), Parkin, and FBX07, which has provided an opportunity to dissect the molecular basis of ubiquitin signaling in PD pathomechanism and develop novel therapeutic strategies in PD(Walden and Muqit, 2017). These findings provided insights into the PCBP-1’s novel roles in differential splicing modulation by protein ubiquitination.

WW Domain Containing E3 Ubiquitin Protein Ligase 2 (*WWP-2*) has been linked to endothelial injury and vascular remodeling as a key regulatory factor (Watt et al., 2018). WWP-2 knockout mice show significantly enhanced angiotensin II (AngII)-mediated endothelial injury and vascular remodeling following endothelial injury (Zhang et al., 2020). The ubiquitin ligase activity of *WWP-2* might be exploited as the potential target for therapeutic intervention to control iron uptake in diseases involving iron dysomoeostasis (Foot et al., 2008). The Ras Homolog Family Member T1 (*RHOT-1*) protein is not only the substrate for the PINK1-PRKN-dependent degradation pathway but also the active modulator of mitophagy. Thus, *RHOT-1* is assumed to be a potential risk factor in PD (Safiulina et al., 2019). Grossmann et al. (2020) has found that RHOT-1’s disease-causing variants are involved in mitochondrial dysfunction, impaired endoplasmic reticulum-mitochondrial tethering, and calcium homeostasis in PD. Thus, this study brought RHOT-1 in the central highlight for potential therapeutic strategies for PD.

Our results showed that *WWP-2*’s alternative 5′splice event was increased. In contrast, cassette exon and alternative 3′splice events were decreased for *RHOT-1* and *EEA-1* genes. Furthermore, *EEA-1* is the marker of endocytosis, which is involved in the recycling of synaptic vesicles and neurotransmitter receptors in neuron (Delaney, 2017) and is critically involved in maintaining protein homeostasis and neuronal health in PD.

Finally, the CLIP-seq approach identified PCBP-1 bound mRNA targets. The ABLIRC algorithm showed that significant fractions of PCBP-1 bound target peaks were enriched at 3′ UTR region and within coding DNA sequences. In particular, PCBP-1 overexpression might regulate the splice junctions by binding at the intergenic and intronic regions, essential sites for AS events, in Jurkat T cells. Based on the GO and KEGG analyses, PCBP-1 could be involved in multiple cellular processes, including gene expression regulation, RNA splicing, and metabolism, as well as protein ubiquitination. These observations might broaden our understanding of the PCBP-1’s physiological mechanisms in the pre-mRNA splicing regulation.

However, our work has some limitations. Firstly, we only explored the role of PCBP1 by overexpression in PC12 in the physiological state, and although some of the differential genes and AS events associated with PD were identified, the expression of the genes in the physiological condition is substantially different from the pathological condition, thus the results of this study need to be further validated in the PD model. Second, only overexpression intervention was applied in this study, making the findings less persuasive. Down-regulation of PCBP1 expression using knockdown or silencing techniques is needed to evaluate the changes in downstream genes and AS events. Finally, there are few overlapping genes between the results of this study and the CLIP-seq data downloaded from GEO, which may be related to species genetic differences.

## Conclusion

Here, we showed that PCBP-1 partially modulates differential mRNA splicing of genes involved in PD pathophysiology in PC12 neuronal cells. The direct modulation of the gene expressions associated with neuroinflammation, protein ubiquitination, and endocytosis had also been demonstrated. Further studies should be conducted to reveal the biological implications of PCBP-1-modulated neuroinflammation, protein ubiquitination, and endocytosis in neurodegenerative conditions. Hence, we provided novel insights that PCBP-1 potentially participated in various types of AS events of genes having crucial roles in PD pathophysiology.

## Data Availability Statement

The datasets presented in this study can be found in online repositories. The names of the repository/repositories and accession number(s) can be found below: https://www.ncbi.nlm.nih.gov/, GSE196153.

## Ethics Statement

The animal study was reviewed and approved by the Ethics Committee of Social Work Department of People’s Hospital of Xinjiang Uygur Autonomous Region.

## Author Contributions

AY drafted the article. AY and HL revised it critically for important intellectual content. CY and SZ contributed to the revision of the manuscript and figure illustrations. SJ and LY made substantial contributions to the conception and design of the review and gave final approval of the version to be published. All authors contributed to the article and approved the submitted version.

## Conflict of Interest

The authors declare that the research was conducted in the absence of any commercial or financial relationships that could be construed as a potential conflict of interest.

## Publisher’s Note

All claims expressed in this article are solely those of the authors and do not necessarily represent those of their affiliated organizations, or those of the publisher, the editors and the reviewers. Any product that may be evaluated in this article, or claim that may be made by its manufacturer, is not guaranteed or endorsed by the publisher.
