# Supplementary figures and images for: PCBP-1 Regulates the Transcription and Alternative Splicing of Inflammation and Ubiquitination-Related Genes in PC12 Cell
Source: Front Aging Neurosci. 2022 Jun 20;14:884837. doi: 10.3389/fnagi.2022.884837 (PMC9251440; doi:10.3389/fnagi.2022.884837)

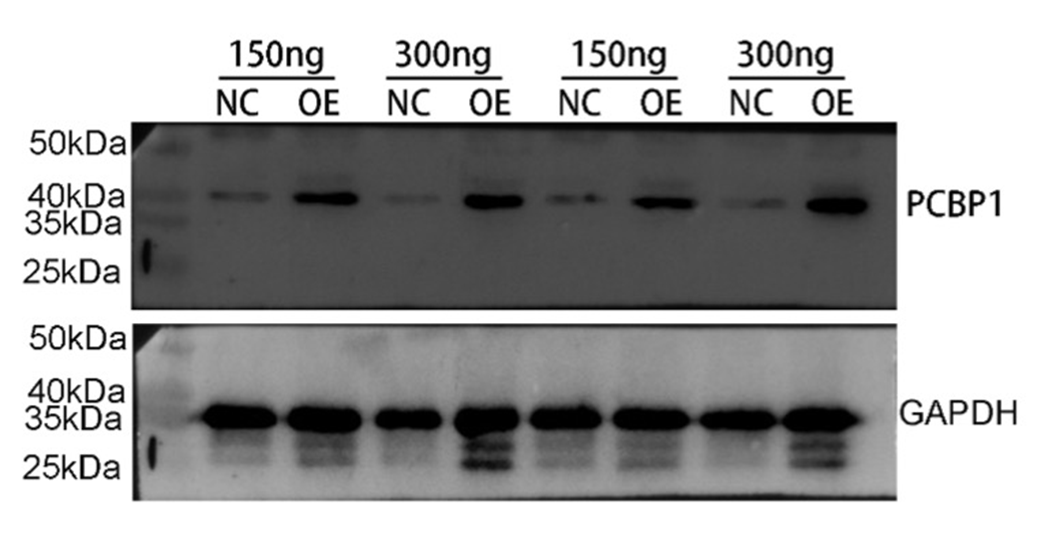

Supplement: Supplementary file 1 [file Image_1.TIFF]
