# Supplementary material for: PCBP-1 Regulates the Transcription and Alternative Splicing of Inflammation and Ubiquitination-Related Genes in PC12 Cell
Source: Front Aging Neurosci. 2022 Jun 20;14:884837. doi: 10.3389/fnagi.2022.884837 (PMC9251440; doi:10.3389/fnagi.2022.884837)
Supplement: Supplementary file 2 [file Table_1.DOCX]

Supplementary Material

# Supplementary Table1 qRT-PCR primers for DEG and AS.

| Gene | Primer | Sequence (5'-3') |
| --- | --- | --- |
| **DEG primers** |  |  |
| GAPDH(Rat) | Forward | TCTCTTGTGACAAAGTGGACA |
|  | Reverse | CCCATTCTCAGCCTTGACTGT |
| PCBP1 | Forward | CCACCTCTAGACGCCTACTC |
|  | Reverse | GTGCATCATGGCAAAGTGAG |
| Ep300 | Forward | TTGACCTGGAACATGACTTA |
|  | Reverse | AGACCTTAGCAATTCTGACA |
| Oas1a | Forward | AGGAACTCAGGAGCACCCC |
|  | Reverse | TGGACAGTATCTCGGAAGCA |
| Mx1 | Forward | CAACGTGCATGTAATGGTT |
|  | Reverse | TGAGGTAAGGCTGTGGAA |
| Mx2 | Forward | GCCCTGTCCTTTCCTGTG |
|  | Reverse | AATGGGGAGAAGTTTCTGGT |
| Lcn2 | Forward | TACAGGGTGACTTTGAAGTA |
|  | Reverse | CTCAGATACAGAGCTACGAT |
| Lfit3 | Forward | AGCGGTTGGTTGAAGATG |
|  | Reverse | TGTCAGCCTCTCCTGTATT |
| Bcl7a1 | Forward | CTCATCAGCCTTGGCTTC |
|  | Reverse | TGATAACAGCAACCAGAGC |
| Bcl7a2 | Forward | CACTTGTAGATTCGTAGGGATG |
|  | Reverse | GAAATGGGTGACCGTTGG |
| **AS primers** |  |  |
| Rhot1-M |  | CCATGTACCCGCACGTGACA |
| Rhot1-AS |  | TTCTTAACAGGCACGTGACA |
| Rhot1-M/AS-R |  | GCAAAGACAGTAGCACCA |
| Wwp2-M |  | CCTCTACCAGTCTTCGAGTGC |
| Wwp2-AS |  | AAATGTGGAATCTTCGAGTGC |
| Wwp2-M/AS-R |  | CCCAAGGGATCATGGTCAG |
| Eea1- M/AS |  | GATTCATCAGCAACTCCT |
| Eea1-AS-R |  | GACTTCTTTTTCTCTGAAGAAC |
| Eea1-M -R |  | AGATGAAACCCTCTGAAGAAC |
| Cutc - M |  | TTCCACAGAGATCCAATGGCA |
| Cutc -AS |  | ATGGTTCAAGATCCAATGGCA |
| Cutc -M/AS -R |  | GGTCTGTGAGCTGCTTTATG |
| Rab1a - M/AS |  | ATCTCCAGCCCAGACCAT |
| Rab1a -AS-R |  | AGCACACATACCAGATCACTG |
| Rab1a -M -R |  | AAGGTGACACCAGATCACTG |
| Traf3-M/AS |  | CAGCAGCATCAGGTCAGA |
| Traf3-AS-R |  | AAAGTCTTACTAGCAGGGAG |
| Traf3-M -R |  | ACGTGCAGGGCCTAGCAGGGAG |

[**Supplementary Table 2 PCBP1-OE versus Ctrl DEGs**](Supplementary%20table%202.xls)**(See Excel File Supplementary table 2)**

[**Supplementary Table 3. Alternative splicing events**](Supplementary%20table%203.xlsx)**(See Excel File Supplementary table 3)**

[**Supplementary Table 4. Alternative splicing events**](Supplementary%20table%204.xlsx)**(See Excel File Supplementary table 4)**
